# Bioactive compounds from marine algae targeting MMP-9: A computational approach to rheumatoid arthritis treatment

**DOI:** 10.1097/MD.0000000000050518

**Published:** 2026-09-04

**Authors:** Abdullah R. Alanzi, Hattan A. Alharbi

**Affiliations:** a Department of Pharmacognosy, College of Pharmacy, King Saud University, Riyadh, Saudi Arabia.

**Keywords:** ADMET, biomarker, MD simulation, MMP-9, molecular docking, rheumatoid arthritis

## Abstract

Rheumatoid arthritis (RA) is a chronic autoimmune systemic inflammatory condition that significantly damages bone and cartilage, leading to severe joint deformities. Matrix metalloproteinase(s) (MMP-9) plays a significant role in both the pathogenesis and management of RA, and its status as a biomarker and a target for inhibition is important in understanding and treating the disease. This work aims to examine the interactions between the molecules of MMP-9 and bioactive compounds derived from marine algae using molecular docking and absorption, distribution, metabolism, excretion, and toxicity investigations. Docking results were assessed using Glide gscore, the top 5 compounds (6,6-Bieckol, Dieckol, Lophocladine B, Sargachromanol E, and Porphyran) docked against protein receptors were chosen. The selected compounds had binding affinities that varied from -8.523 to -6.327 kcal/mol, and the selected compounds also had undergone absorption, distribution, metabolism, excretion, and toxicity analysis. Density functional theory analysis was conducted against finalized compound, Lophocladine B, with HOMO-LUMO gaps of 4.235 and 5.275 eV, respectively, confirming electronic stability and binding-relevant reactive sites consistent with its predicted MMP-9 inhibition. It was discovered that only Lophocladine B met the selection criteria, so this compound was subjected to molecular dynamics simulations for stability analysis. Moreover, the docking studies were supported by redocking validation of the native inhibitor, heavy-atom root mean square deviation, benchmarking with a known inhibitor, and molecular dynamics simulations along with a replication run. The results of the study imply that these substances might ultimately be employed to treat rheumatoid arthritis. Although this study only included computer validations, in vitro investigations are needed to convert these potential inhibitors into medicinal products.

## 1. Introduction

A persistent inflammatory condition called rheumatoid arthritis (RA) is characterized by the immune system destroying synovial joints. If it progresses and is not treated properly, it can cause substantial disability and early mortality. RA, in conjunction with extra-articular symptoms and comorbidities, increases mortality. Increased mortality is caused by RA, as well as extra-articular symptoms and comorbidities. Reducing inflammation, preventing joint degeneration, and maintaining functional capacity are the major objectives of RA therapy.^[1–3]^

A family of Zn2 + dependent endopeptidases known as matrix metalloproteinases (MMPs) actively contributes to the breakdown of extracellular matrix constituents. It has been demonstrated that they are crucial for the invasion, destruction, and creation of bone erosion of cartilage.^[4]^ It is produced in the joint capsule primarily by macrophages and monocytes; chondrocyte production seems to be negligible.^[5]^ In 1960, MMPs, were identified. There are now 25 distinct MMPs known, with 24 of them being found in humans.^[6]^

Matrix Metalloproteinase-9 (MMP-9) belongs to the gelatinases subgroup and can degrade gelatin. MMP-9 gene is one of the most studied MMP-9s genes, and it has been linked to RA disease activity. In RA, MMP-9 contributes to joint destruction. In humans, it is located on chromosome 20. It consists of 13 exons and 2 unique domains that are important in TIMP (tissue inhibitor of metalloproteinases) binding: hemopexin domains and fibronectin-like domains.^[7–9]^ The fibronectin-like domain contributes to the breakdown of the extracellular matrix (by being necessary for binding to denatured collagen or gelatin.^[10–12]^ When comparing the synovial fluid of inflamed joints between patients with osteoarthritis and those with RA, high levels of MMP-9 were discovered. This implies that although both disorders are chronic types of arthritis, RA’s joint destruction is associated with elevated levels of pro-inflammatory MMP-9, which in turn leads to enhanced synovial fibroblast migration, invasion, survival, and proliferation. Given its importance in joint damage and inflammation, MMP-9 is a potential therapeutic target for the development of drugs that specifically inhibit its activity. MMP-9 inhibitors have been studied to slow disease progression, reduce pain, and raise living standards generally in RA patients.^[4,12–15]^

The catalytic zinc ion in the conserved HExGHxxGxxH motif is coordinated by 3 histidine residues and is also polarized by an adjacent glutamate residue, which activates a water molecule bound to the zinc ion to cleave the scissile peptide bond of gelatin and denatured collagen.^[16]^ It is not expressed constitutively, but rather is induced by the inflammatory environment of the rheumatoid synovium, in which pro-inflammatory cytokines like TNF-α, Interleukin-1 beta and Interleukin-17 activate the NF-κB and activator protein 1 signalling pathways, leading to the transcriptional upregulation of MMP-9 in synovial fibroblasts, macrophages and infiltrating neutrophils.^[17]^ MMP-9 is activated to degrade type IV and type V collagen of the basement membrane, to promote invasion of pannus and migration of leukocytes, and to release matrix-bound VEGF, which stimulates synovial angiogenesis, thus linking extracellular-matrix turnover to the self-perpetuating inflammation and bone erosion characteristic of RA.^[18]^ Importantly, broad-spectrum, zinc-chelating MMP inhibitors, like the hydroxamate marimastat, were not developed clinically because of dose-limiting musculoskeletal syndrome caused by nonselective inhibition of the MMP family, and this has led to the development of agents that are more selective, binding to the less-conserved S1′ specificity pocket, rather than the catalytic zinc itself.^[19]^ This unmet need is the basis for the evaluation of structurally diverse metabolites derived from marine algae as potential selective MMP-9 binders, which is an under-exploited chemical space compared to synthetic hydroxamates.^[20]^

Although the conventional approaches to developing new medications are costly and time-consuming, advancement in technology have resolved these shortcomings.^[21–25]^ The usefulness of using computational techniques to identify small compounds in the medication development process has increased recently. The study of interactions between small compounds and target protein active sites has made extensive use of molecular docking.^[26,27]^ This study intends to investigate the molecular interactions between MMP-9 and marine algae-derived bioactive compounds using molecular docking and absorption, distribution, metabolism, excretion, and toxicity (ADMET) analyses.

## 2. Methodology

### 2.1. Preparation of ligand

The structures of the bioactive compounds that were extracted from marine algae targeting Phaeophyceae were obtained using PubChem (https://pubchem.ncbi.nlm.nih.gov/). The LigPrep tool from Maestro was used to prepare the phytochemical structures.^[28]^ During this process, ionization states were generated at a target pH of 7.0 ± 2.0 using Epik; appropriate tautomers were enumerated, and stereoisomers arising from unspecified chiral centers were generated up to a maximum of 32 per ligand, while specified chiralities in the input structures were retained. Each ligand’s geometry was optimized, resulting in the generation of 32 conformers. A single low-energy ring conformation was generated for each structure, and the resulting 3-dimensional geometries were energy-minimized using the empirical OPLS_2005 force field.^[29]^ Counter-ions and salts were stripped from 3D conformers of the compounds.

### 2.2. Molecular docking studies

The prepared bioactive compounds were used in the docking of the MMP-9 receptor. The X-ray crystallographic structure of MMP-9 was retrieved from the Protein Data Bank Database (ID: 4H3X) and prepared for the docking using the Protein Preparation Wizard.^[27,30]^ During receptor preparation, several stages were done including the generation of disulfide bonds, assignment of zero-order metal bonds, and addition of hydrogens. The additional ligands and crystal water were also removed. In the optimization step, the pKa values of ionizable groups were optimized at pH 7.0 utilizing the PROPKA program.^[31]^ Finally, the OPLS_2005 force field was used for energy minimization. After the protein preparation, a 3-dimensional grid was constructed at Cartesian coordinates with values of 10.47, 7.44, and 8.48 for X, Y, and Z, respectively, for site specific docking. The compounds were docked to the protein using the SP mode of Glide.^[32]^ Further, the ADMET profiles and physicochemical properties of the docked compounds were analyzed by the QikProp tool.^[33]^

### 2.3. Molecular dynamics simulation

The complexes were analyzed for protein conformation and ligand stability by running a simulation of 500 ns by using GROMACS.^[34]^ Chemistry at HARvard Molecular Mechanics 36 force field was used for protein and ligand parameterization. For ligand parameterization, bond-charge-increment charges were used with a net charge of 0.000. For Zinc paramterization, a nonbonded (electrostatic) model with a single ZN2 moleculetype was used. Topology files were generated with formal charge + 2.0, mass 65.37, standard Chemistry at Harvard Molecular Mechanics Zn LJ. The systems were solvated by placing them in a cubic box of 10 Å, filled with the TIP3P water model.^[35]^ 2fs of timestep was used for the production run. Cutoffs were used such as r_vdw_switch 1.0 → r_vdw 1.2 nm, Verlet scheme, nstlist 20, rlist 1.2 nm. A linear constraint solver algorithm on H-bonds was used for constraints. To replicate physiological conditions, counter ions were added for neutralization, and 0.15 M NaCl salt was incorporated. The temperature and pressure of the system were set to 310K v-rescale thermostat and 1 atm with a c-rescale barostat, respectively by using the NPT ensemble. After a relaxation phase of 5000 steepest-descent minimization to equilibrate the system, the production run was started to record the trajectories after 50 ps time interval. The GROAMCS built-in commands were used to analyze the trajectories and perform root mean square deviation (RMSD), root mean square fluctuation (RMSF), SASA, H-bonds, Rog analyses to test and evaluate the stability of the biomolecular structures.

## 3. Results

### 3.1. Molecular docking

The crystal structure of the human MMP-9 catalytic domain was chosen because it is in a pharmacologically relevant conformation, bound to the broad-spectrum hydroxamate 10B (biphenylsulfonyl acetohydroxamic acid, “CC27”) in the active site, and has a high resolution (1.76 Å). The catalytic zinc (ZN301, coordinated by the His226/His230/His236 triad and located 1.85-2.0 Å from the hydroxamate) was retained in the receptor, whereas the second (structural) Zn^2+^, all 3 structural calcium ions, crystallization additives (glycerol, PEG, propanediol) and all water molecules were removed. The catalytic zinc’s coordination was treated non-covalently, without explicit metal-ligand bonds. The compounds were prepared, and their docking study was conducted against the MMP-3 protein. The binding affinities of all docked compounds were analyzed, and then the top 5 compounds with binding affinities ranging from -8.523 to -6.327 kcal/mol were selected for further analysis (Table 1). The docking scores of the selected compounds suggested that these have potential for inhibiting the function of the MMP-9 protein.

**Table 1:**
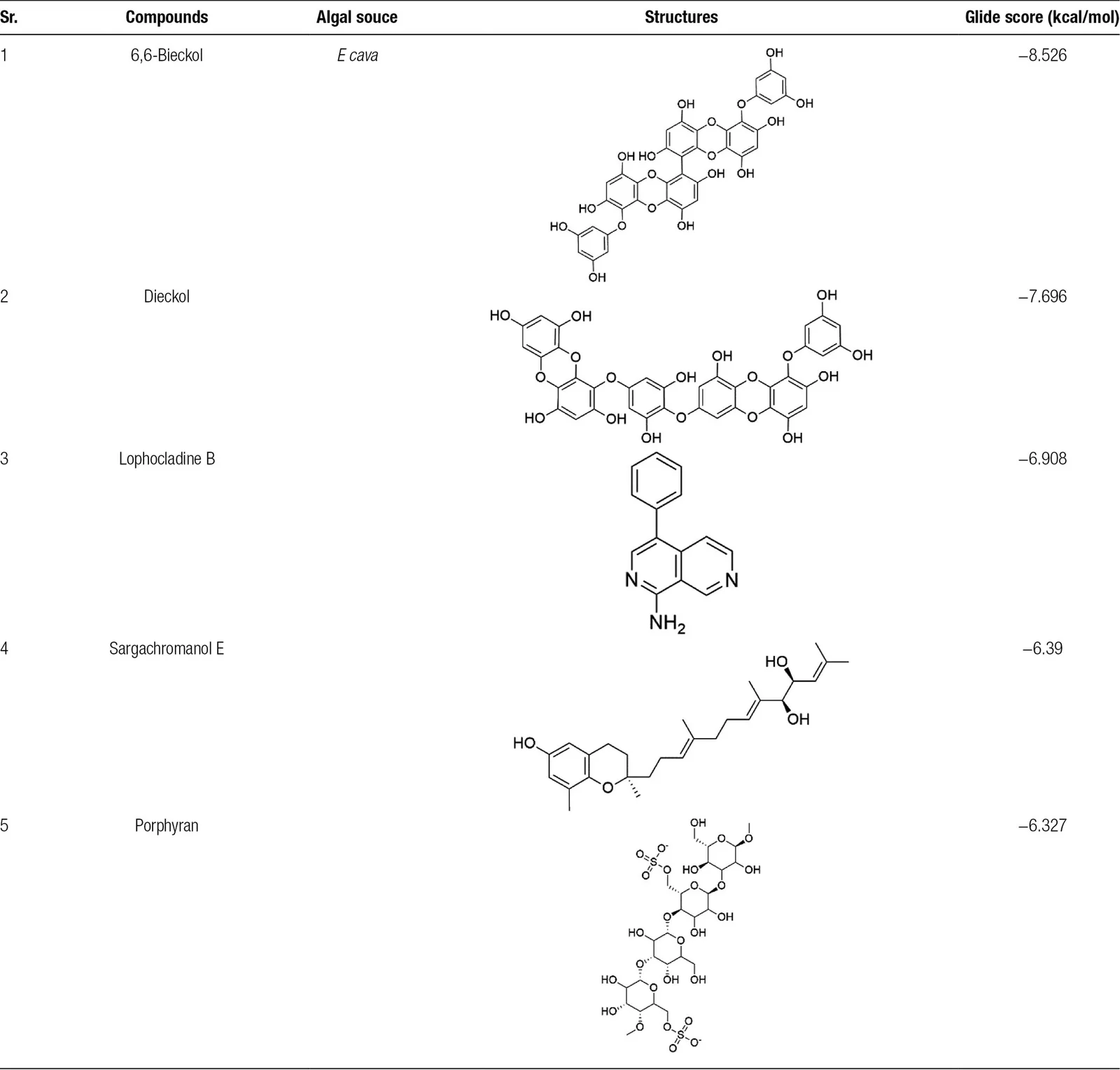
The glide scores of the docked compounds against MMP-9 protein.

### 3.2. Post docking analysis

**Figure 1.**
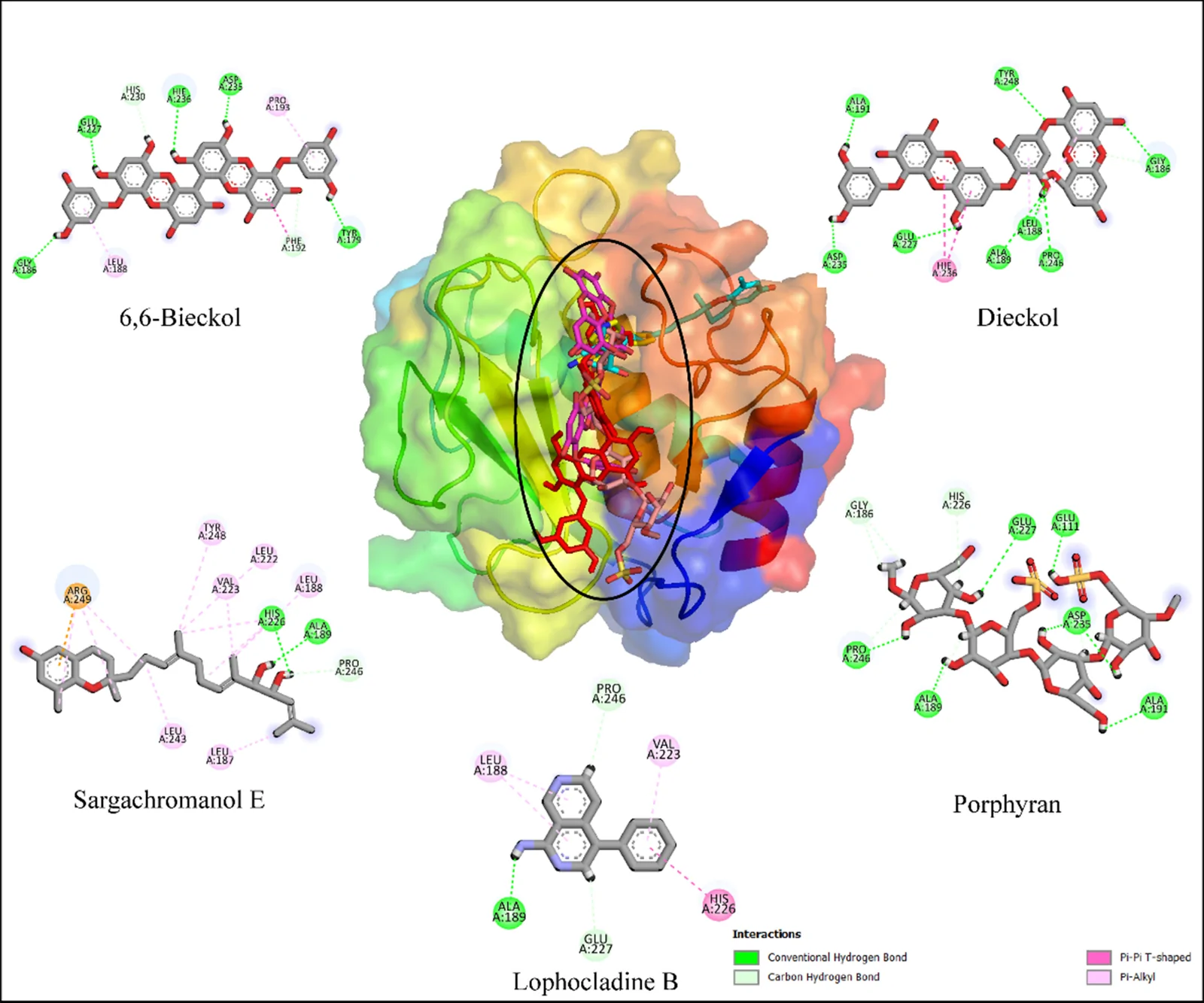
The interactions of the selected compounds with MMP-9 protein. MMPs = matrix metalloproteinase(s).

The docked poses of the selected compounds were analyzed by the Discovery Studio client tool to find the molecular interactions. The molecular interactions mainly involved hydrogen bonding, van der Waals interactions, pi-sigma interactions, and alkyl (hydrophobic) interactions. The molecular interactions of each candidate compound help in determining the binding affinities. Especially, the hydrogen bonds among ligand and protein atoms play an important role in the strength of the protein-ligand complex.^[36]^ **6,6-Bieckol** 5 conventional hydrogen bonds with Gly186, Glu227, His236, Asp235, Tyr179, 2 carbon hydrogen bonds with His230, Phe192, and 2 alkyl interactions with Leu188, Pro193. **Dieckol** formed 8 conventional hydrogen bonds with Asp235, Glu227, Ala191, Ala189, Leu188, Pro246, Gly186, Tyr248 and 1 alkyl interaction with His236. Similarly, **Lophocladine B** made 1 conventional hydrogen bond with Ala189, 2 carbon hydrogen bonds with Pro246 and Glu227, and 3 alkyl interactions with Leu188, Val223, and His226. **Sargachromanol E** made 2 conventional hydrogen bonds with Ala189, His226, 1 carbon hydrogen bond with Pro246, 1 Pi-cation interaction with Arg249, and 6 alkyl interactions with Leu187, Leu243, Val223, Tyr248, Leu222, Leu188. Lastly, **Porphyran** 6 forms conventional hydrogen bonds with Ala189, Pro246, Asp235, Ala191, Glu111, Glu227 and 2 carbon hydrogen bonds with Gly186, His226 (Fig. 1). The lengths of hydrogen bonds and interactions are shown in Table 2.

**Table 2:** The molecular interactions and bond lengths of the selected compounds with MMP-9 protein.

Sr.	Compounds	Interactions and bond lengths
1	6,6-Bieckol	**Conventional Hydrogen Bond:** Gly186(2.26), Glu227(1.65), His236(2.53), Asp235(1.69), Tyr179(2.09) **Carbon Hydrogen Bond:** His230(2.58), Phe192(2.70) **Alkyl:** Leu188(4.26), Pro193(4.82)
2	Dieckol	**Conventional Hydrogen Bond:** Asp235(1.63), Glu227(2.73), Ala191(1.71), Ala189(2.83), Leu188(2.18), Pro246(2.87), Gly186(2.38), Tyr248(2.61) **Alkyl:** His236(4.98)
3	Lophocladine B	**Conventional Hydrogen Bond:** Ala189(1.73) **Carbon Hydrogen Bond:** Pro246(3.03), Glu227(2.51) **Alkyl:** Leu188(4.27), Val223(5.33), His226(3.79)
4	Sargachromanol E	**Conventional Hydrogen Bond:** Ala189(2.29), His226(1.95) **Carbon Hydrogen Bond:** Pro246(2.69) **Pi-Cation:** Arg249(3.40) **Alkyl:** Leu187(4.80), Leu243(5.38), Val223(3.94), Tyr248(5.38), Leu222(5.41), Leu188(4.30)
5	Porphyran	**Conventional Hydrogen Bond:** Ala189(1.78), Pro246(2.05), Asp235(1.83), Ala191(2.06), Glu111(1.49), Glu227(2.48) **Carbon Hydrogen Bond:** Gly186(2.32), His226(2.67)

### 3.3. Binding pose analysis

**Figure 2.**
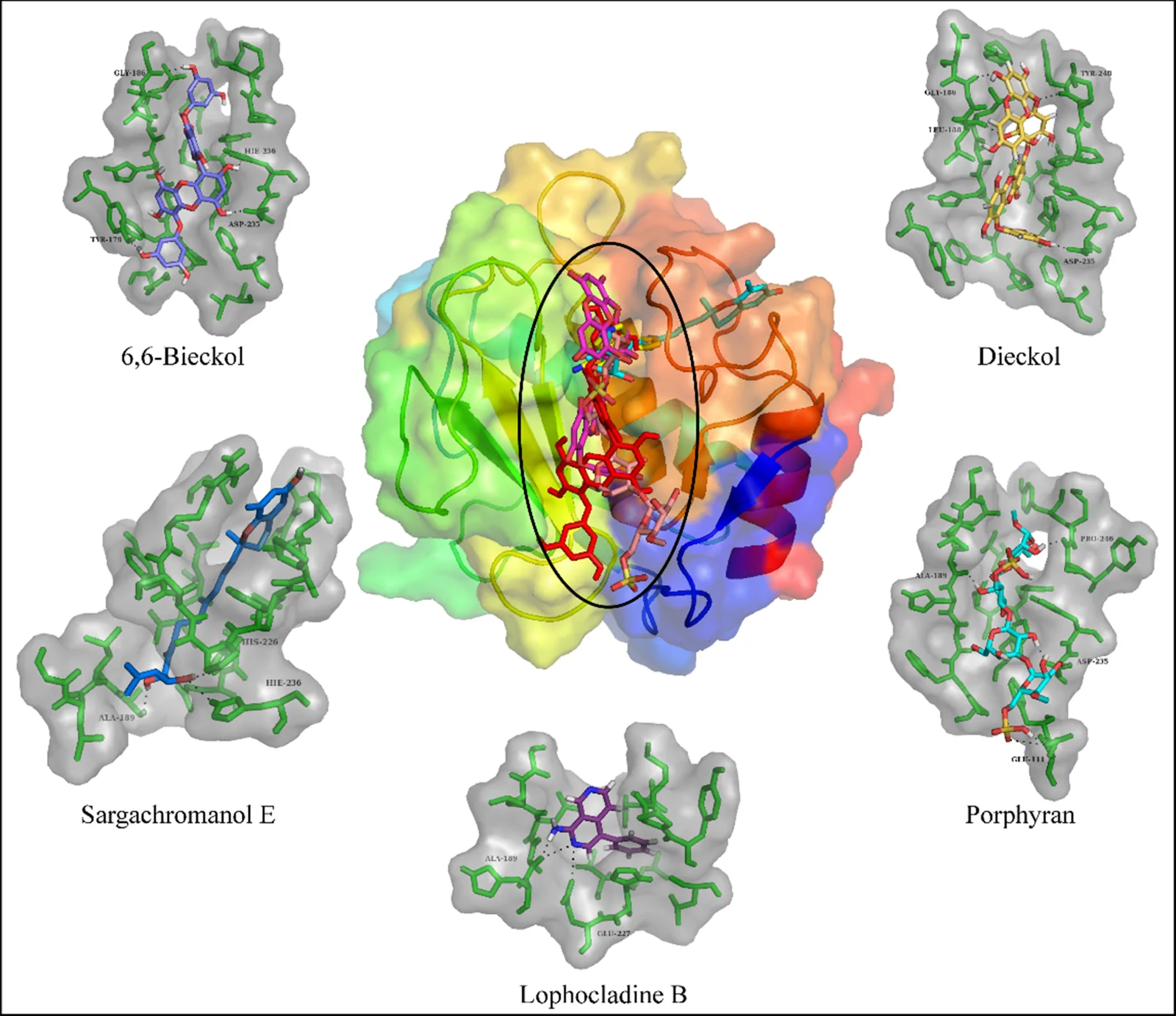
The binding modes of the selected compounds. The residues forming hydrogen bonds with the compounds are labeled.

The plausible binding modes of the selected compounds were observed by alignment with the co-crystal ligand. This alignment showed that the docked poses of the hits occupied the same space in the binding sites of the MMP9 as occupied by the co-crystal ligand (Fig. 2).

**Figure 3.**
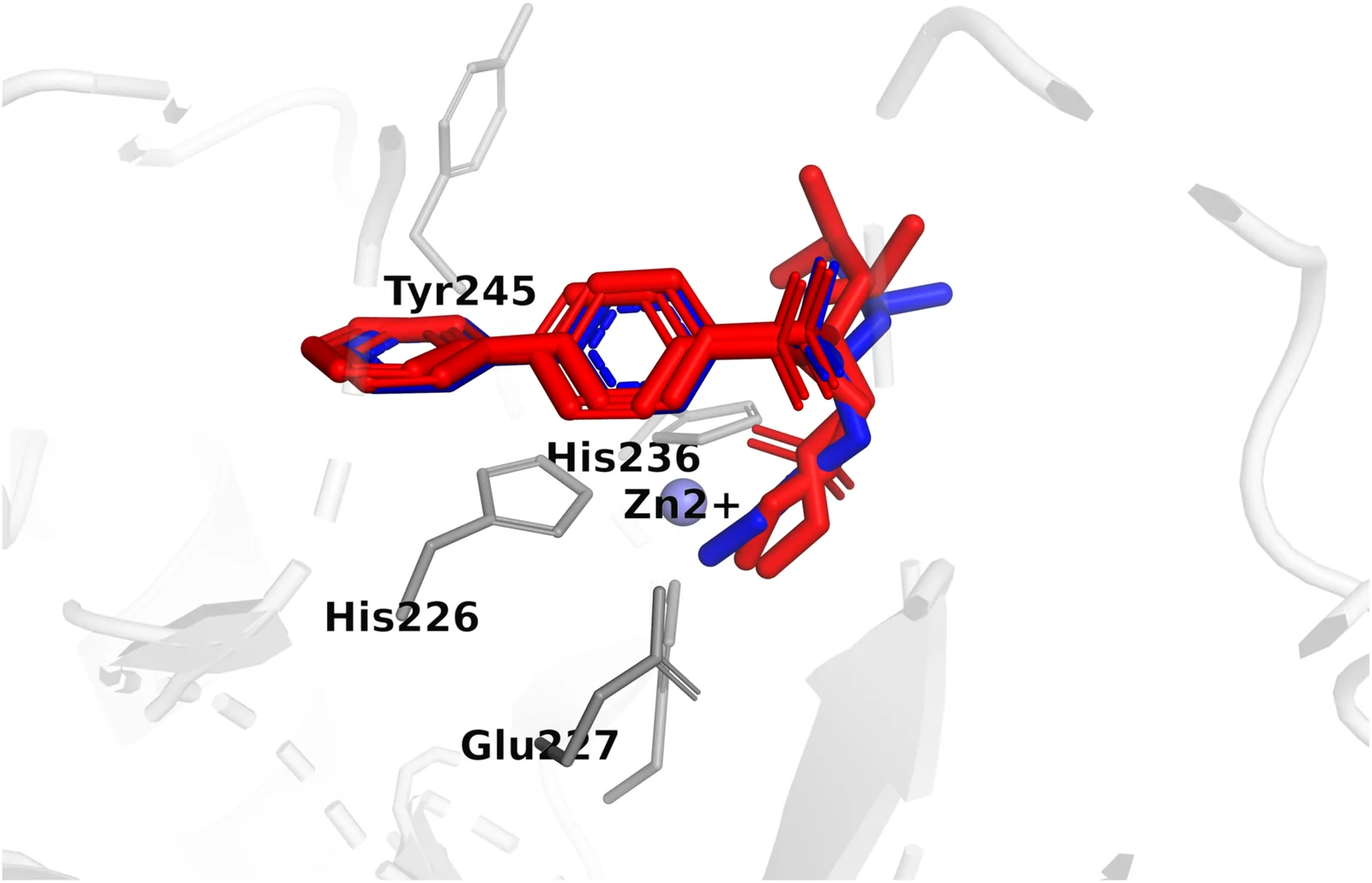
Redocking validation of the docking protocol with the native ligand 10B of MMP9’s protein (PDB: 4H3X). MMPs = matrix metalloproteinase(s), PDB = Protein Data Bank.

The docking protocol was validated by re-docking the co-crystallized inhibitor 10B into the prepared MMP-9 catalytic domain (chain A of 4H3X, catalytic Zn^2+^ retained), which reproduced the crystallographic binding mode with a symmetry-corrected heavy-atom RMSD of 1.04 Å for the top-ranked pose (mode 1) and 0.88 Å for mode 4, both well below the accepted 2.0 Å threshold. As an additional benchmark, the clinically evaluated broad-spectrum MMP inhibitor marimastat was docked under identical conditions and correctly recovered the canonical binding signature, positioning its hydroxamate 1.85 Å from the catalytic zinc with contacts to the His226/His230/His236 triad and Glu227. Against this validated framework, Lophocladine B (-8.2 kcal/mol) occupied the same S1′ specificity pocket as 10B and scored more favorably than marimastat (-6.4 kcal/mol), confirming good shape complementarity to the active site while underscoring that Vina scores rank pocket fit rather than absolute potency (Fig. 3).

### 3.4. ADMET analysis

The ADMET profiles of the compounds were examined by the QikProp tool, which showed that the compounds have properties within the acceptable range of parameters (Table 3). The cutoff values for the parameters were as follows: “QPlogHERG (<-5), QPlogPo/w (-2.0 to 6.5), QPlogBB (-3.0 to 1.2), QPPCaco (<25 poor, >500 great), and QPlogKhsa (-1.5 to 1.5).” Upon analysis of the ADMET properties, it was observed that only **Lophocladine B** and **Sargachromanol E** met the selection criteria, so these 2 compounds were subjected to molecular dynamics (MD) simulations for stability analysis.

**Table 3:** The ADMET and physicochemical characteristics of the compounds.

Compounds	MW	HBD	HBA	QPlogPo/w	QPlogHERG	QPPCaco	QPlogBB	QPlogKhsa
6,6-Bieckol	742.5	12	18	0.621	−7.383	0.087	−6.579	−0.287
Dieckol	742.5	11	18	1.162	−6.997	0.632	−5.144	−0.268
**Lophocladine B**	**221.26**	**1**	**3**	**2.187**	−**5.068**	**978.435**	−**0.464**	−**0.066**
Sargachromanol E	428.6	3	4	5.837	−5.865	750.446	−1.51	1.162
Porphyran	852.7	10	27	−7.575	−2.209	0.002	−8.428	−4.193

### 3.5. Density functional theory

The optimized geometries, frontier molecular orbitals and global reactivity descriptors of the 2 lead compounds, Lophocladine B, was characterized using density functional theory (Fig. 3). Lophocladine B was found to adopt a planar naphthyridine-phenyl framework, and to have a moderate HOMO-LUMO gap of 4.235 eV (E_HOMO = −5.6651 eV, E_LUMO = −1.4298 eV), indicating significant intramolecular charge transfer with the HOMO localized on the naphthyridine core and the LUMO delocalized over the phenyl ring. The resulting descriptors confirmed these trends: Lophocladine B was seen to be the softer, more electrophilic compound (η = 2.118 eV, ω = 2.971 eV). For Lophocladine B compound, the MEP maps localized negative potential over the heteroatom (ring-nitrogen or oxygen) centers and positive potential over the N-H/O-H hydrogens, identifying complementary hydrogen-bonding sites, while the density of states profiles confirmed well-separated occupied and virtual manifolds and the reduced density gradient, / Non-covalent interaction(s), analyses indicated that intramolecular stabilization is dominated by weak van der Waals interactions with localized steric regions at the ring centers.

### 3.6. MD simulation

#### 3.6.1. RMSD

**Figure 4.**
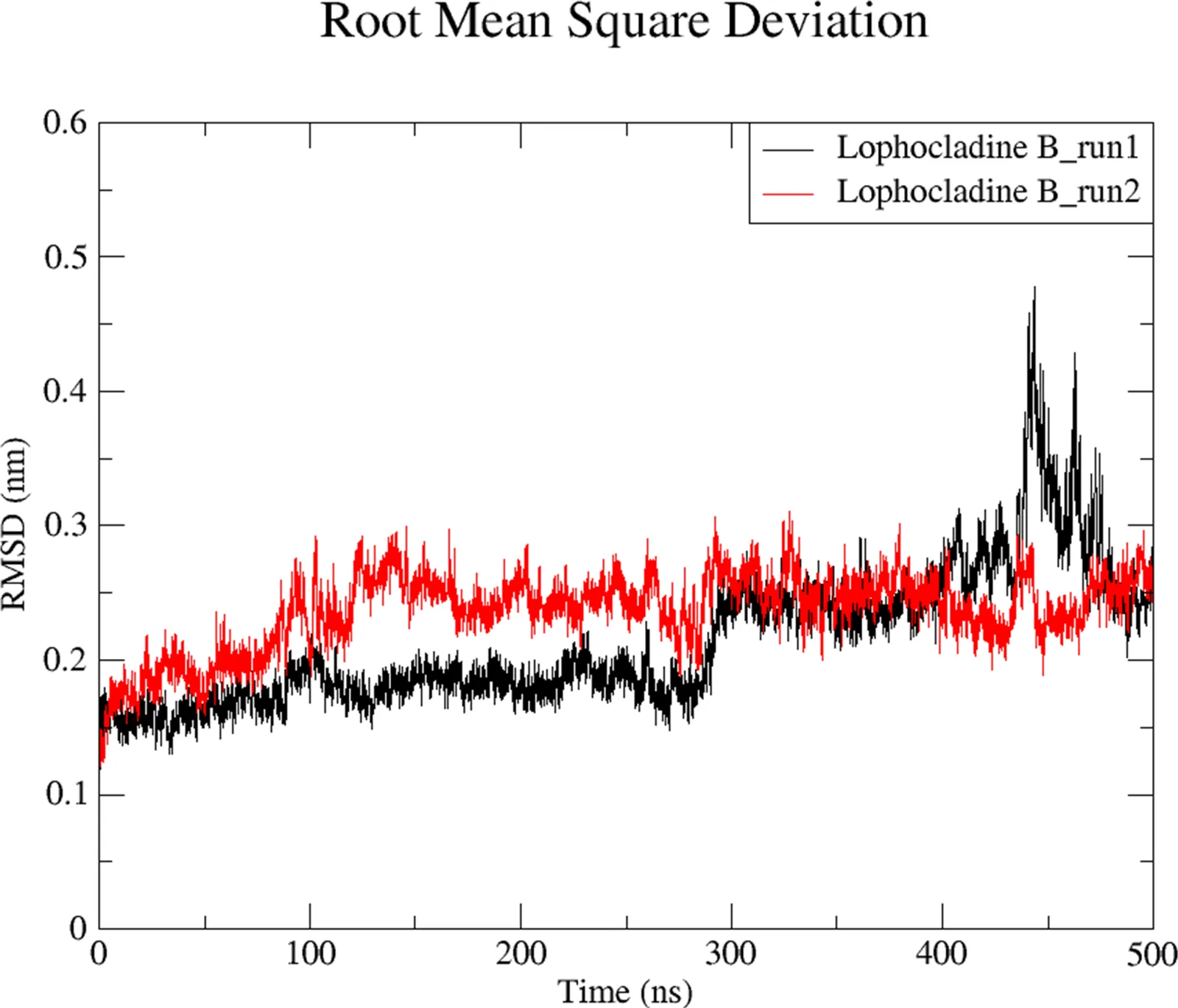
The RMSD of MMP-9 complexes calculated during a 500 ns simulation for Lophocladine B run1 and run2. MMPs = matrix metalloproteinase(s), RMSD = root mean square deviation.

To confirm the stability of the protein-ligand complexes, MD simulations of 500ns were performed in 2 independent replicates to investigate the binding sites of the selected Lophocladine B compound against the MMP-9 receptor. The RMSD of the Cα atoms was calculated in order to look into the complexes’ deviations and general structural changes during the simulation.^[37]^ The Lophocladine B complex maintained a stable receptor backbone in both replicates, equilibrating within the first ~50 ns and plateauing thereafter at 0.214 ± 0.052 nm (run1) and 0.237 ± 0.029 nm (run2), with maximum excursions of only 0.478 and 0.311 nm, respectively; the close agreement between the 2 independent runs confirms that the MMP-9-fold is not perturbed by ligand binding. The two replicates differed in ligand behavior: in run1 the ligand remained continuously seated in the S1′ pocket (ligand RMSD 0.160 ± 0.087 nm overall; 0.168 ± 0.031 nm over the final 50 ns), whereas in run2 it underwent a transient partial unbinding event during the first ~120 ns (peak ligand RMSD 5.22 nm at ~7 ns) before reseating into the same pocket and remaining stably bound for the remaining ~380 ns (0.335 ± 0.018 nm after 450 ns). The spontaneous return of the ligand to the S1′ site in run2 indicates that this pose is thermodynamically preferred rather than a docking artifact; accordingly, molecular mechanics/generalized born surface area energies for run2 were computed over the post-reseating window to exclude the unbound frames (Fig. 4).

#### 3.6.2. RMSF

**Figure 5.**
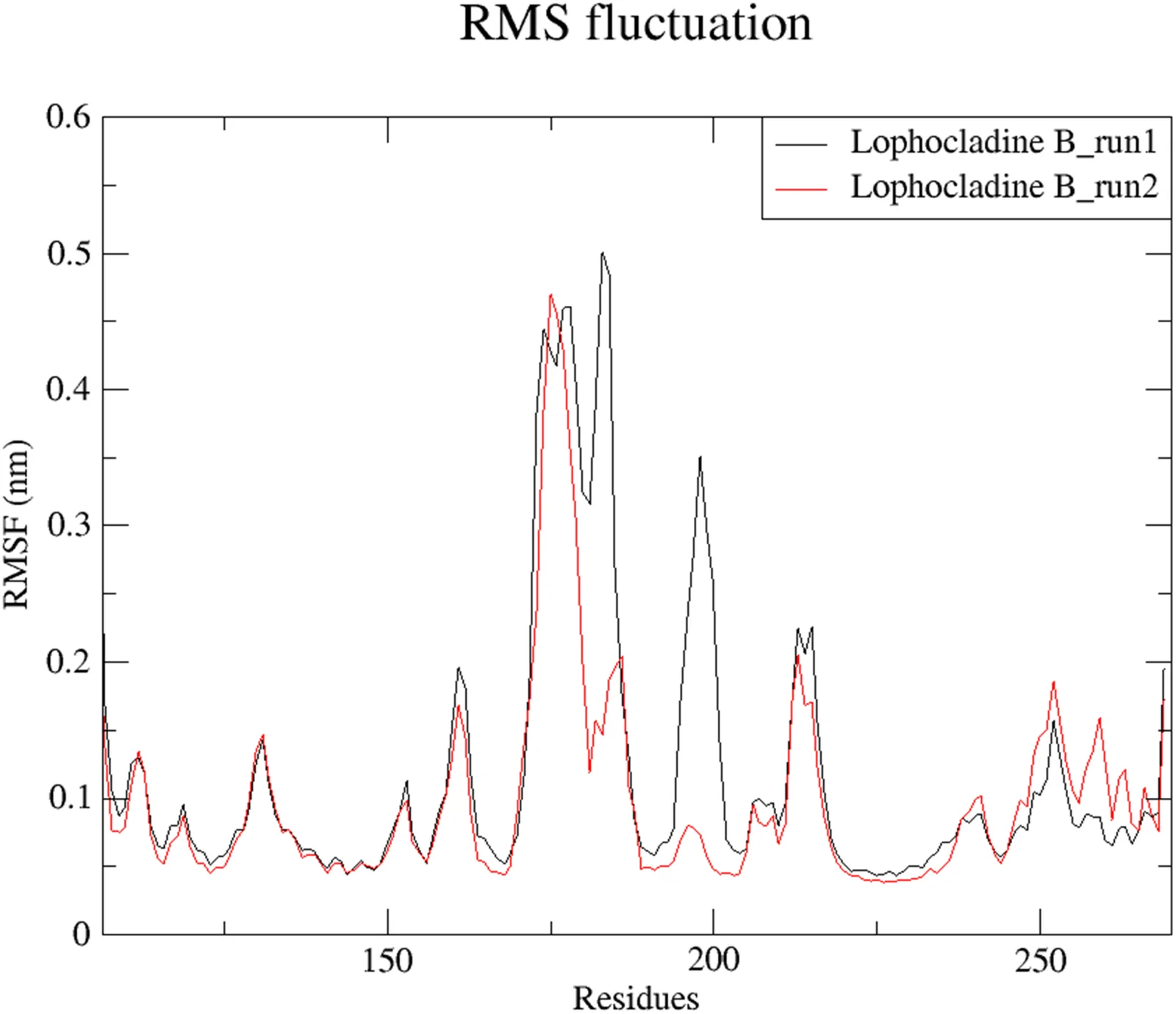
The comparative RMSF plots of the MMP-9 protein for both complexes using C-alpha atoms of the receptor. MMPs = matrix metalloproteinase(s), RMSF = root mean square fluctuation.

RMSF values were calculated to investigate the protein residues’ dynamics upon interacting with the ligands.^[38]^ Both profiles share the expected shape: high mobility at the N-terminus (residues ~50-80, peaks ~0.6-0.8 nm) and C-terminus (~0.7 nm), low in the structured core. The dominant feature in both runs is a single well-defined peak over the 173-184 surface loop, reaching 0.501 nm at Arg183 in run1 and 0.470 nm at residue 175 in run2; the reproducibility of this peak across 2 independent trajectories confirms it as an intrinsic property of the MMP-9 catalytic domain rather than a ligand-induced effect, and it lies remote from the S1′ binding site. The RMSF plots revealed that most of the residues did not show many fluctuations during simulation as the RMSF values were lower than 0.15 nm (1.5 Å) for 80% of residues in run1 and 85% in run2 throughout the simulation with overall mean fluctuations of 0.119 ± 0.102 nm (run1) and 0.098 ± 0.074 nm (run2), indicating that the ligands did not exert the fluctuations in the protein. In contrast, the residues in the loop regions showed high fluctuations in the presence of the compound reaching around 0.50nm (5Å), suggesting increased flexibility in these regions (Fig. 5). Critically, the active-site residues remained markedly rigid in both replicates: the catalytic zinc-binding triad His226, His230 and His236 together with Glu227 fluctuated by only 0.038-0.066 nm, and the S1′ lining residues Leu187/Leu188, Val223, Leu243, Tyr245, Met247 and Tyr248 stayed below 0.11 nm, consistent with a stably occupied and conformationally restrained binding pocket. According to the RMSF analysis, the protein-ligand complex exhibited overall stability, as most residues maintained a rigid conformation.

#### 3.6.3. Radius of gyration and hydrogen bonding analysis

**Figure 6.**
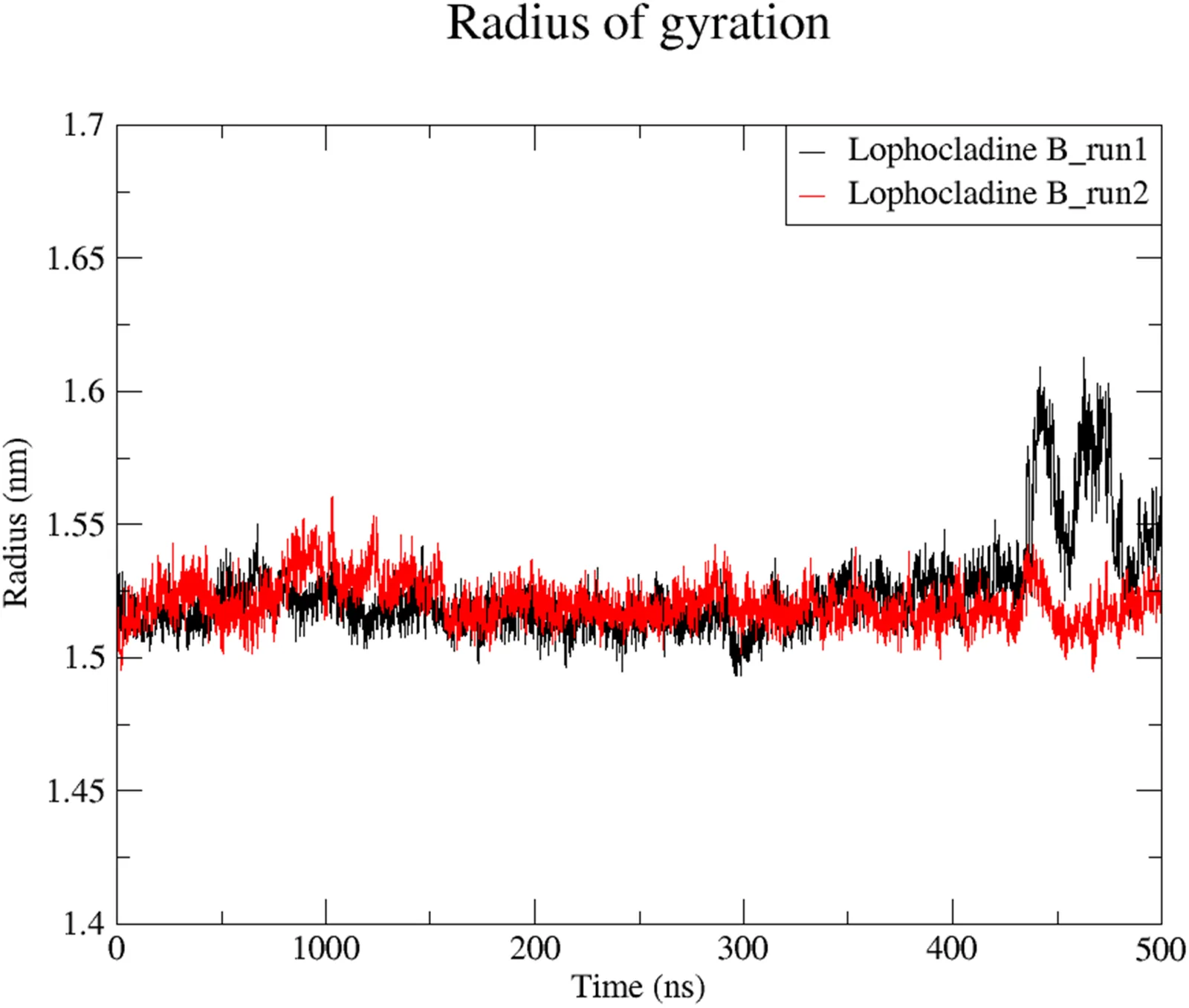
Radius of gyration profiles for both complexes.

**Figure 7.**
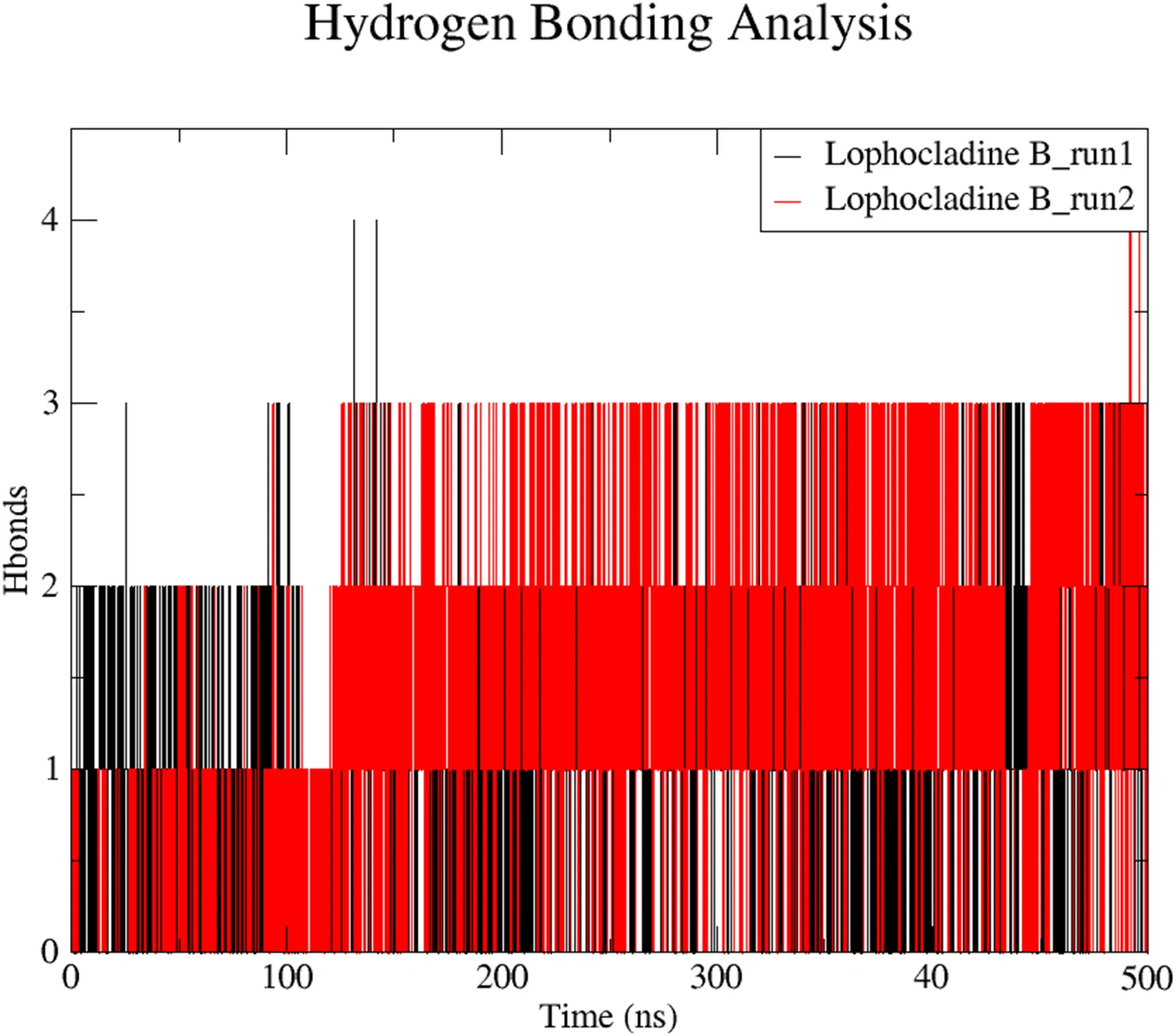
Hydrogen bonding profiles of Lophocladine B run1 and run2 with the MMP9 receptor. MMPs = matrix metalloproteinase(s).

The radius of gyration and hydrogen bonding analysis among the atoms of the protein and ligands were observed during the simulation. The Lophocladine B complex remained highly compact and essentially invariant throughout both replicates, with mean radius of gyration values of 1.524 ± 0.017 nm (run1) and 1.521 ± 0.008 nm (run2) and total drift confined to 1.49 to 1.61 nm; the agreement between the 2 independent runs to within 0.003 nm demonstrates that no domain opening, expansion or unfolding occurred over 500 ns and that the MMP-9 catalytic domain retained its native globular fold upon ligand binding (Fig. 6). The ligand formed persistent rather than transient hydrogen bonds with the receptor: at least 1 protein–ligand hydrogen bond was present for 73 % of frames in run1 and 75 % in run2, with mean occupancies of 0.97 ± 0.75 and 1.31 ± 0.98 bonds and maxima of 4 bonds in both runs. Restricting run2 to the post-reseating window (>120 ns) raises this to 1.64 ± 0.85 bonds with ≥1 bond present in 91% of frames, compared with 1.03 ± 0.77 bonds and 77% occupancy for the equivalent window of run1, confirming that the rebound pose is at least as well hydrogen-bonded as the continuously bound pose and reinforcing the RMSD-based conclusion that the S1′ site is the preferred binding mode (Fig. 7**).**

## 4. Discussion

Matrix metalloproteinase are the primary enzymes in the pathophysiology of cartilage and joint deterioration, and they are being considered as potential markers of the early erosive phase of rheumatoid arthritis (RA).^[36]^ MMP-9 is an enzyme that is a member of the matrix metalloproteinase family, which is important for tissue remodeling and extracellular matrix component degradation. MMP-9 is overproduced in RA, which leads to joint damage and inflammation by accelerating the deterioration of cartilage and joint tissues. The degree and activity of the disease are correlated with its levels. MMP-9 level monitoring can be used as a biomarker to evaluate the course of the disease and the effectiveness of treatment. Elevations of MMP-9 are linked to more severe inflammation and joint damage, which makes it a valuable marker to help direct treatment choices.^[4,12,39,40]^

The current research aims to use ADMET and molecular docking analysis to study the interactions between the molecules of compounds produced from marine algae and the MMP-9 protein. Therapeutically valuable chemicals of significant importance, such as peptides, proteins, fatty acids, amino acids, polysaccharides, sterols, phenolic compounds, oligosaccharides, vitamins, photosynthetic pigments, and minerals, have been abundantly available because of algae biotechnology.^[41,42]^ Furthermore, a wide range of potential health advantages and uses for bioactive molecules and/or compounds derived from algae exist. These include antiviral, antibacterial, anticancer, antifungal, anti-inflammatory, antidiabetic, anti-obesity, antioxidant, and biofertilizer compounds.^[42]^ So far, thousands of compounds obtained from marine species have undergone screening and been employed for many medicinal applications.^[43]^ Marine algae-derived compounds have gained attention in the field of medical research due to their potential anti-inflammatory and antioxidant properties. While not extensively studied as MMP-9 inhibitors for RA therapy, some marine algae compounds show promise in modulating inflammatory pathways and may indirectly affect MMP-9 activity.^[44]^

The structures of bioactive compounds extracted from marine algae were obtained from PubChem. The prepared compounds were docked to the prepared MMP-9 receptor to predict binding affinities using the Glide tool’s standard precision mode. A computer method used in molecular biology and drug development is called “molecular docking,” to anticipate and analyzes interactions between a small molecule (ligand) and a target macromolecule (receptor), which is often a protein or nucleic acid. The basic purpose of molecular docking is to anticipate the ligand’s most energetically favorable binding mode within the receptor’s binding site.^[45]^ This understanding is essential for comprehending how potential drug molecules engage with their target proteins, which can affect the development of new therapies and the enhancement of existing ones.^[46,47]^ The top 5 compounds (6,6-Bieckol, Dieckol, Lophocladine B, Sargachromanol E, and Porphyran) were chosen for further investigation based on their binding affinities. The binding affinities of these selected compounds ranged from −8.523 to −6.327 kcal/mol. These binding affinities indicate that the compounds may have the potential to inhibit the function of the MMP-9 protein. The molecular interactions among the compounds and the protein were observed, which involved mainly hydrogen bonding and several alkyl interactions. These interactions play a significant role in determining the binding efficacy of compounds to the protein.^[48]^

Mechanistically, the residues that recur in the top-ranked complexes (His226, His230, His236, Glu227 and Asp235) are associated with the catalytic apparatus of MMP-9, where the His226/His230/His236 triad coordinates the active-site zinc ion, and Glu227 is the catalytic base in the conserved HExGHxxGxxH motif. The binding of these residues suggests that the marine scaffolds enter directly into the catalytic cleft and may prevent the positioning of the substrate around the zinc center, but do not bind at a distant surface, which is essential for true enzymatic inhibition.^[16]^ Notably, the present ligands make these contacts primarily via hydrogen bonding and hydrophobic or π-interactions, rather than direct zinc chelation, which is mechanistically significant because the clinical failure of hydroxamate-based, zinc-chelating MMP inhibitors has been attributed to poor selectivity across the MMP family, and non-chelating chemotypes that exploit the surrounding S1′ pocket are now preferred for selective gelatinase inhibition.^[20,49]^ The 2 leads further illustrate complementary pharmacophoric strategies: Lophocladine B is a compact, planar naphthyridine alkaloid with a charge-transfer-active framework that is well suited for directional hydrogen bonding and π-stacking. This chemotype diversity, 2 structurally unrelated scaffolds converging on the same catalytic site, further underscores the importance of these compounds as independent starting points for the design of selective MMP-9 inhibitors and makes marine secondary metabolites a viable and yet underutilized source of leads for targeting metalloproteinase compared to traditional synthetic libraries.^[50]^

Additionally, ADMET analysis was performed on the selected compounds. The ADMET evaluation, a crucial phase in the drug development process, assesses a compound’s potential for use in medicine. It is feasible to predict a proposed drug’s level of toxicity, behavior, and effect in the human body by looking at the ADMET features. To detect and reduce any drug-related risks, ADMET testing is crucial.^[51–53]^ When the ADMET properties were examined, it was discovered that only Lophocladine B met the selection criteria, so these 2 compounds were subjected to MD simulations for stability analysis. Lophocladine B was prioritized as the sole lead based on the convergence of a favorable docking score (-6.908 kcal/mol) with a broadly drug-like ADMET profile, satisfying the recommended ranges for QPlogPo/w, QPlogBB, QPPCaco and QPlogKhsa. Its QPlogHERG value (−5.068) fell only marginally past the −5.0 concern threshold, in contrast to Sargachromanol E’s more pronounced deviation (−5.865), making Lophocladine B the more defensible candidate to carry forward despite both compounds otherwise meeting the same permeability and distribution criteria. We treat this marginal hERG signal as a flag rather than a cleared liability, and recommend experimental hERG screening alongside in vitro MMP-9 inhibition assays before further development of this compound.

The density functional theory analysis confirms the docking and MD results, as both leads have electronically stable scaffolds, with Lophocladine B having a narrower HOMO-LUMO gap (4.235 eV), which suggests higher polarizability and charge-transfer capacity. The frontier-orbital distributions are informative in terms of the mechanism: the charge-transfer active naphthyridine-phenyl system of Lophocladine B with the MMP-9 active site. The MEP maps further rationalize the binding behavior, as the electronegative ring-nitrogen/oxygen centers and the positive N-H/O-H hydrogens are aligned with the hydrogen-bond donor–acceptor contacts (e.g., with Ala189 and Glu227) observed in the simulations. The complementary reactivity profiles, 1 favoring polar charge-transfer engagement and the other favoring stability with hydrophobic, dispersion-driven contacts along its terpenoid chain, suggest that both are electronically viable MMP-9 inhibitors and confirm their stability in the dynamics analysis.

Taken together, the Lophocladine B complex shows the opposite global behavior high RMSD and an expanded radius of gyration, yet its per-residue RMSF is lower, a pattern most consistent with large-scale rigid-body or domain motions (the protein moving as relatively intact units that inflate whole-structure RMSD without raising local fluctuations) rather than local unfolding. Because both ligands sustain only weak, intermittent hydrogen bonding, neither is anchored primarily by H-bonds, suggesting that any binding stability is driven more by hydrophobic/shape complementarity in the pocket than by a specific polar contact network.^[47]^ MD simulation studies have demonstrated that these compounds have shown stability as potent inhibitors inside the protein binding pocket. Therefore, comprehending the underlying molecular processes of protein and ligand interaction may prove to be highly beneficial in the field of drug therapy to control RA. New drug formulations and inhibitors are in the process of being developed; further experimental research is necessary to validate these therapeutic targets.

## 5. Conclusion

The present study intended to perform molecular modeling approaches on marine algae derived compounds to target the MMP-9 protein. The molecular docking and MD simulation results show that 2 marine algae compounds, Lophocladine B, have the capacity to function as anti-RA agents. These 2 candidates emerged from the convergence of docking, ADMET profiling, and dynamics, being distinguished by favorable binding affinities, drug-like ADMET profiles, and complexes that remained stable across 500 ns of simulation with an independent replication run. Mechanistically, both compounds anchor to key MMP-9 active-site residues such as Ala189 and Glu227 through hydrogen bonding and hydrophobic contacts, providing a consistent structural basis for their inhibitory potential. Density functional theory -based frontier orbital, MEP, and non-covalent interaction(s), analyses corroborated the electronic stability and binding-relevant reactivity of Lophocladine B, reinforcing its potential as a marine algae-derived MMP-9 inhibitor. However, computer validations were described here, and more in vitro and in vivo research is required before these potential inhibitors can be turned into therapeutic drugs.

## Acknowledgments

The authors would like to express their appreciation to Ongoing Research Funding program (ORF-2026-885) at King Saud University, Riyadh, Saudi Arabia, for supporting this research.

## Author Contributions

**Conceptualization:** Abdullah R. Alanzi.

**Data curation:** Abdullah R. Alanzi.

**Methodology:** Hattan A. Alharbi.

**Validation:** Hattan A. Alharbi.

**Writing – original draft:** Abdullah R. Alanzi, Hattan A. Alharbi.

**Writing – review & editing:** Abdullah R. Alanzi, Hattan A. Alharbi.
